# Prophylactic Management of Vestibular Migraine: A Systematic Review

**DOI:** 10.1002/acn3.70234

**Published:** 2025-10-30

**Authors:** Hussain Ali Almohammed, Husna Irfan Thalib, Kawthar Faisal Kushara, Bushra Wadi Bin Saddiq, Bayan Mohammed Khair Al Zoabi, Tanveer Nidal Khan, Ahmed Salah Morad, Rimas Warid Aljuaid, Lamyaa Mohammed Gharawi, Shahad Jawdat Alsaygh, Mable Pereira, Shaima Abuhulayqah

**Affiliations:** ^1^ College of Medicine King Faisal University Al Ahsa Saudi Arabia; ^2^ General Medicine Practice Batterjee Medical College Jeddah Saudi Arabia; ^3^ College of Medicine King Abdulaziz University Jeddah Saudi Arabia; ^4^ College of Medicine Taif University Taif Saudi Arabia; ^5^ School of Medicine Lincoln American University Georgetown Guyana; ^6^ Neurology Department King Fahad Hospital Al Ahsa Saudi Arabia

**Keywords:** anti‐CGRP monoclonal antibodies, beta‐blockers, prophylactic treatment, vestibular migraine, vestibular rehabilitation

## Abstract

**Background:**

Vestibular migraine (VM) is a neurological disorder characterized by episodic vertigo in patients with migraine, contributing to substantial functional impairment. Although VM is relatively common, a consensus on effective prophylactic treatments is lacking. This systematic review aimed to evaluate the efficacy and safety of pharmacological and adjunct non‐pharmacological interventions for VM management.

**Methods:**

The systematic review was conducted following the PRISMA 2020 guidelines. PubMed, Web of Science, and Google Scholar were searched from inception to April 2025. Randomized controlled trials or cohort (retrospective and prospective) studies assessing prophylactic pharmacological or adjunct non‐pharmacological management in adults diagnosed with VM were included. The risk of bias was evaluated using the Cochrane RoB 2 and MINORS tools.

**Results:**

Nine studies (three RCTs, six cohort studies; total *n* = 687) met the inclusion criteria. Beta‐blockers, particularly propranolol, and antiepileptics, such as topiramate, consistently reduced VM symptoms, as measured using validated scales (Dizziness Handicap Inventory, Vertigo Symptom Scale, and Vestibular Activities of Daily Living). Anti‐CGRP monoclonal antibodies (erenumab and galcanezumab) showed favorable efficacy and tolerability. By contrast, calcium channel blockers and antidepressants yielded inconsistent outcomes. Vestibular rehabilitation, when used as an adjunct therapy, demonstrated functional benefits. Reported adverse effects were generally mild and treatment specific. Substantial heterogeneity in treatment protocols, outcome assessments, and follow‐up intervals precluded meta‐analysis; therefore, findings were synthesized qualitatively.

**Conclusion:**

Moderate‐quality evidence supports the use of propranolol and topiramate as first‐line prophylactic treatments for VM. Emerging data suggest that anti‐CGRP agents may be beneficial for refractory cases. Larger, rigorously designed trials are necessary to establish standardized and individualized treatment protocols.

## 1 Introduction

Vestibular migraine (VM) is a neurological disorder characterized by episodic vertigo in individuals with a history of migraine. First clearly described by Boenheim in 1917, this disorder has been referred to over the decades by various terms, including “migrainous vertigo,” “migraine‐associated vertigo,” “migraine‐associated dizziness,” and “migraine‐related vestibulopathy” [1, 2]. The evolving nomenclature reflects both advancements in understanding the disorder's pathophysiology and the complexity of its clinical presentation.

From an epidemiological perspective, VM is common, affecting approximately 3% of the general population. It ranks as the second most frequent cause of dizziness and is the leading cause of spontaneous (non‐positional) episodic vertigo [3, 4, 5, 6]. Despite its prevalence, VM remains underdiagnosed, primarily due to benign findings on physical examination, symptom overlap with other vestibular disorders, and the absence of specific biomarkers.

Diagnostic criteria for VM were formalized by the Bárány Society Committee for the Classification of Vestibular Disorders in collaboration with the Migraine Classification Subcommittee of the International Headache Society. Before establishing the diagnosis, clinicians must thoroughly evaluate the cause of headache, given the symptomatic overlap with other disorders.

A diagnosis of VM requires the following:

1. At least five episodes of vestibular symptoms ranging from moderate to severe in intensity, lasting from 5 to 72 h.
2. A current or history of migraine with or without aura.
3. At least half of vertigo episodes must be accompanied by one or more migraine‐associated features, including:
  - Photophobia
  - Phonophobia
  - Visual aura
  - Headache with at least two of the following characteristics:
    - Unilateral location
    - Pulsatile quality
    - Moderate to severe intensity
    - Worsened by physical activity
4. Symptoms must not be accounted for by any other disorder [7].

Although the pathophysiology of VMs remains incompletely understood, several mechanisms have been proposed. One proposed theory attributes the symptoms to transient hypoperfusion of the inner ear due to vasospasm during a migraine attack, which could lead to vertigo and, in some cases, sudden sensorineural hearing loss [8]. A more substantiated hypothesis posits that sensitization and activation of the trigeminovascular system trigger the release of pro‐inflammatory neuropeptides, such as substance P and calcitonin gene‐related peptide (CGRP). These neuropeptides interact with cortical and subcortical structures, including the thalamic and vestibular cortices, which are involved in nociceptive and vestibular processing. Neuroimaging studies have demonstrated structural and functional alterations in these pathways, further supporting the notion of dysfunction within the vestibulothalamocortical pathway in patients with VMs [9, 10, 11].

Pharmacological management of VMs is divided into abortive and prophylactic strategies. Abortive treatments aim to alleviate acute symptoms and include typical migraine medications such as triptans, antihistamines like diphenhydramine and meclizine, and antidopaminergic agents such as metoclopramide. Prophylactic therapy focuses on reducing the frequency and severity of future attacks, thereby significantly improving quality of life. Options for pharmacological prophylaxis include beta‐blockers (e.g., propranolol), calcium channel blockers (e.g., flunarizine), antiepileptics (e.g., valproic acid and topiramate), antidepressants (e.g., amitriptyline), and CGRP monoclonal antibodies such as erenumab [12, 13]. In addition to medication, non‐pharmacological interventions, such as vestibular rehabilitation therapy (VRT), cognitive‐behavioral therapy, and lifestyle modifications, including stress reduction and proper sleep hygiene, play an important role in comprehensive VM management [13, 14, 15].

In this systematic review, pharmacological and non‐pharmacological prophylactic therapies for VMs were assessed for their efficacy and safety in reducing the frequency and intensity of migraine recurrence.

## 2 Methodology

### 2.1 Protocol Approval, Registration, and Patient Consent

This systematic review followed the recommendations of the Preferred Reporting Items for Systematic Reviews and Meta‐analyses (PRISMA) 2020 guideline checklist [16]. The protocol for the current study was registered in PROSPERO (registration number: CRD420251001816). Ethical approval and informed consent were not required because of the nature of the study.

### 2.2 Search Strategy

The electronic databases PubMed, Web of Science, and Google Scholar were systematically searched to extract all relevant articles published until April 2025. Google Scholar was included to capture grey literature; however, primary reliance was placed on PubMed and Web of Science for peer‐reviewed data. The search combined terms related to (i) the condition: “vestibular migraine,” “migrainous vertigo,” “migraine‐associated dizziness,” “vestibular dysfunction,” “vestibular symptoms”; (ii) interventions: “beta‐blockers,” “antiseizure/antiepileptic medications,” “calcium channel blockers,” “tricyclic antidepressants,” “serotonin‐norepinephrine reuptake inhibitors,” “vestibular rehabilitation,” “physical therapy,” and specific migraine therapeutics (e.g., erenumab, fremanezumab, topiramate, propranolol, venlafaxine); and (iii) study design: randomized or controlled clinical trials, cohort, prospective, or retrospective studies. The complete Boolean search strings for each database, including all synonyms and operators, are provided in Table S1.

### 2.3 Study Selection Criteria

Studies were eligible for inclusion if they were published in English and included patients aged 18 years or older with a confirmed diagnosis of VM. We included studies in which VM was diagnosed according to established criteria. The majority used the International Classification of Headache Disorders, 3rd edition (ICHD‐3), and the Bárány Society criteria [7]. All clinical trials or cohort studies, whether prospective or retrospective, that assessed prophylactic pharmacological and non‐pharmacological therapies for VM compared with placebo or other prophylactic treatments were included. Studies involving pediatric patients or focusing on outcomes that were not of interest were excluded. Additionally, case reports, cross‐sectional studies, case–control studies, opinion papers and editorials, review articles, and incomplete or ongoing clinical trials were excluded.

### 2.4 Screening

Four reviewers (KK, LG, RA, and SA) simultaneously screened all extracted articles. Each reviewer independently performed an initial screening by title and abstract using Rayyan [17]. Articles that met the inclusion criteria were subjected to full‐text screening by the same reviewers. Any disagreements were resolved through discussion.

### 2.5 Data Extraction and Risk of Bias Assessment

Three researchers (ASM, BB, and TK) extracted data from studies that passed both stages of screening. Extracted variables included article title, author names, publication year, publication journal, country of origin, study design, number of participants and their demographics, and outcomes of interest. Any discrepancies were resolved through discussion. The risk of bias was assessed at the outcome level of each study using the Methodological Index for Non‐Randomized Studies (MINORS) and the Cochrane Risk of Bias 2 (RoB 2) assessment for clinical trials [18, 19].

### 2.6 Outcome Measures

The primary outcomes of interest were the efficacy and safety of therapeutic and nontherapeutic interventions for VM based on changes in patient scores on the Vertigo Symptom Scale (VSS), Dizziness Handicap Inventory (DHI), and Vestibular Disorders Activities of Daily Living (VADL) scale. Secondary outcomes determine the best choice for the management of VM and the efficacy of anti‐CGRP in patients with VM.

### 2.7 Data Analysis

A formal meta‐analysis was not feasible due to the heterogeneity of study design, interventions, and outcomes. Following Cochrane SWiM (Synthesis Without Meta‐analysis) guidance, a structured vote‐counting synthesis was conducted using only analyzable pre‐ and posttreatment paired data available in the extraction sheet. For each intervention arm, validated VM outcomes (VSS, DHI, MIDAS) and symptom metrics (frequency, duration, VAS) were screened. If pre‐ and post‐intervention means were extractable, the study was classified as showing *Improvement* (reduction from baseline), *No change*, or *Worsening*. Where outcomes were reported only as posttreatment values, responder proportions, or narrative descriptions, these were considered not analyzable and excluded from the vote‐counting. Intervention arms were grouped by pharmacological class.

## 3 Results

**FIGURE 1:**
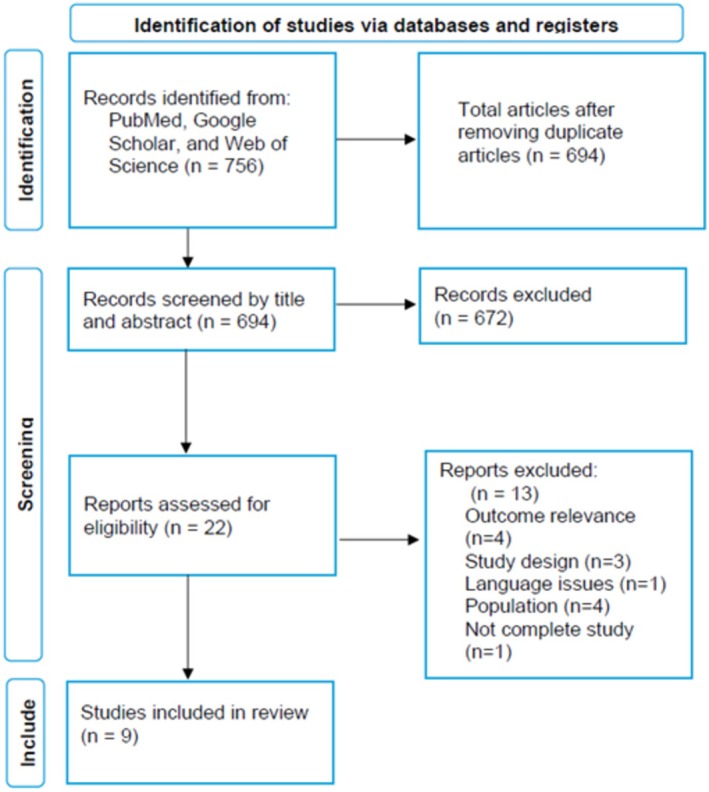
PRISMA flowchart describing the search strategy.

A total of 756 studies were identified through database searches (PubMed, Google Scholar, and Web of Science). After excluding 62 duplicates, 694 records were screened based on titles and abstracts. Of these, 672 were excluded due to irrelevance to the research questions. The remaining 22 full‐text articles were assessed for eligibility. Following full‐text review, 13 studies were excluded for the following reasons: lack of outcome relevance (*n* = 4), incompatible study designs (*n* = 3), language barriers (*n* = 1), mismatched populations (*n* = 4), or incomplete data (*n* = 1). Finally, nine studies met the inclusion criteria and were included in this systematic review (Figure 1).

The demographic and methodological characteristics of the nine studies are presented in Table 1 [20, 21, 22, 23, 24, 25, 26, 27, 28]. The study included 687 participants across studies conducted in eight countries between 2007 and 2024, including six retrospective observational studies and three randomized controlled trials (RCTs). The sample sizes varied considerably, ranging from 23 participants in Lovato et al.'s [26] Swiss cohort to 304 participants in Koc's Turkish trial [28]. A female predominance was consistently observed across all studies, with proportions ranging from 63% to 92.9%. The mean age varied considerably, from 34 years in the youngest cohort to 53.2 years in the oldest. Disease duration exhibited particularly wide variability, with Çelik et al. [22] reporting a mean of 115.1 days (SD 23.6) while Maksoud Nassar et al. [27] documenting durations extending up to 138.4 months (SD 132.1). Comorbidity profiles revealed frequent associations with migraine disorders (75% prevalence in Hoskin and Fife's 2022 US cohort) [20], vestibular dysfunction (35.7%) [20], affective disorders (reaching 39.3% for anxiety/depression) [20], and cardiovascular conditions (31.9% hypertension prevalence in Salmito et al.'s 2017 Brazilian study [23]).

**TABLE 1:** General characteristics of included studies.

Study ID (author, year)	Country	Study design	Blinding	Participants (*n*)	Age (mean ± SD or range)	Sex (female %)	Disease duration (mean ± SD or range)	Comorbidities
Hoskin and Fife, 2022 [20]	USA	Observational (retrospective)	N/A	28	52.2 ± 13.5	92.9%	119.4 months	Migraine (75%), Vestibular disease (35.7%), Depression (21.4%), Anxiety (39.3%)
Iwasaki et al., 2007 [21]	Japan	Observational (retrospective)	N/A	33	40 (18–62)	69.7%	N/A	Migraine with aura (45%)
Çelik et al., 2020 [22]	Turkey	Observational (retrospective)	N/A	38	47.55 ± 13.59	71.1%	115.15 ± 23.60 days	N/A
Salmito et al., 2017 [23]	Brazil	Observational (retrospective)	N/A	47	47.5 ± 10.9	83%	Headache: 10.8 ± 11 years; Vertigo: 6.0 ± 6.5 years	Hypertension (31.9%), Anxiety/Depression (17%), Dyslipidemia (19.1%)
Liu et al., 2017 [24]	China	RCT	Single‐blinded	75	Ven: 53.22 ± 15.55; Flu: 51.45 ± 15.43; Val: 52.35 ± 16.01	69.3% (overall)	N/A	N/A
Baier et al., 2009 [25]	Germany	Observational (retrospective)	N/A	100	Male: 49 (21–71); Female: 46 (25–72)	63%	N/A	N/A
Lovato et al., 2023 [26]	Switzerland	Observational (retrospective)	N/A	23	45.2 ± 7.8	91%	37.9 ± 12.3 months	Unilateral headache (78%), Photophobia/Phonophobia (91%)
Maksoud Nassar et al., 2023 [27]	Egypt	RCT	Open‐label	45	Cin: 37.7; Pro: 38.3; Top: 39 (18–50)	77.8%	Headache: Cin 138.4 ± 132.1; Pro 103.2 ± 83.5; Top 118.0 ± 72.6 months	N/A
Koc, 2024 [28]	Turkey	RCT	Open‐label	304	Cit: 34 (24–58); Amit: 36 (22–56)	75.3%	N/A	N/A

Abbreviations: Amit, amitriptyline; Cin, cinnarizine; Cit, citalopram; Flu, flunarizine; N/A, not available or not applicable; Pro, propranolol; RCT, randomized controlled trial; SD, standard deviation; Top, topiramate; Val, valproate; Ven, venlafaxine; VM, vestibular migraine.

### 3.1 Risk of Bias Assessment

**FIGURE 2:**
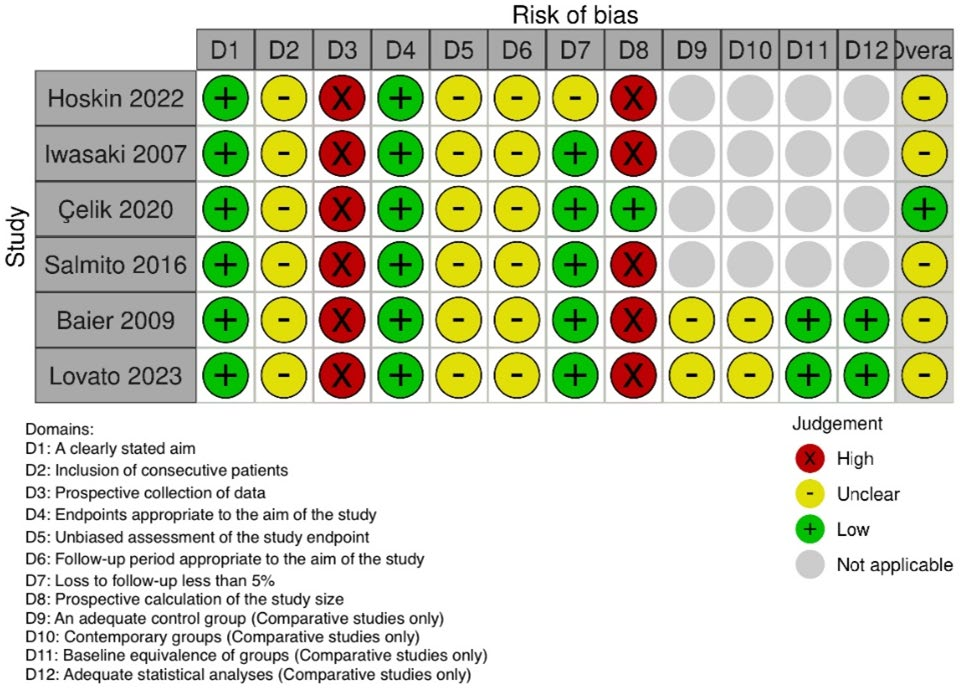
Traffic light plot: RoB assessment using MINORS tool for observational studies (*n* = 6).

**FIGURE 3:**
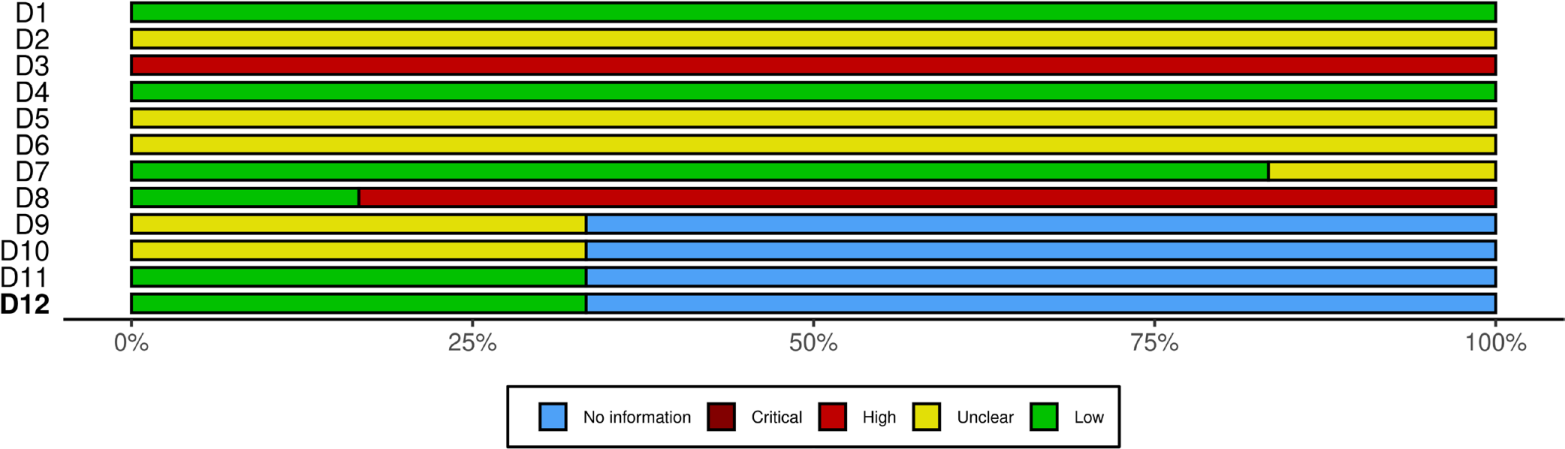
Summary plot: RoB assessment using MINORS tool for observational studies (*n* = 6).

The risk of bias in the six observational studies was assessed using the MINORS tool (Figures 2 and 3). All studies clearly defined their aims (100%), but only Çelik et al. [22] and Lovato et al. [26] reported consecutive patient enrollment, increasing selection bias in the others. All six studies were retrospective, which introduced recall bias, especially for long‐term outcomes. Only Salmito et al. [23] used blinded outcome assessors. The other five studies relied on unblinded treating clinicians, thereby raising the risk of detection bias. Follow‐up reporting was inconsistent. Lovato et al. [26] achieved complete follow‐up, but Iwasaki et al. [21], Baier et al. [25], and Çelik et al. [22] had > 20% loss, reducing data reliability. Overall, MINORS scores showed variations in design quality, endpoint clarity, and follow‐up strength (Figure 3) [25, 26].

**FIGURE 4:**
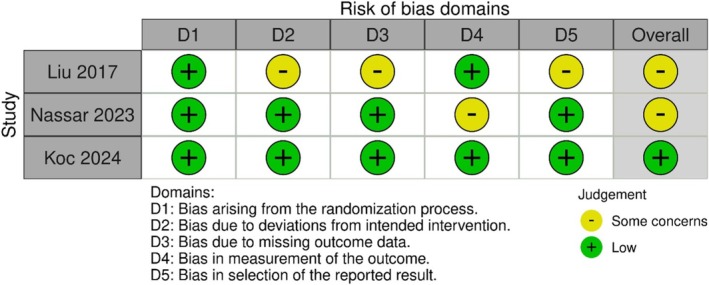
Traffic light plot: RoB assessment using Cochrane Rob2 tool for randomized controlled trial (*n* = 3).

**FIGURE 5:**
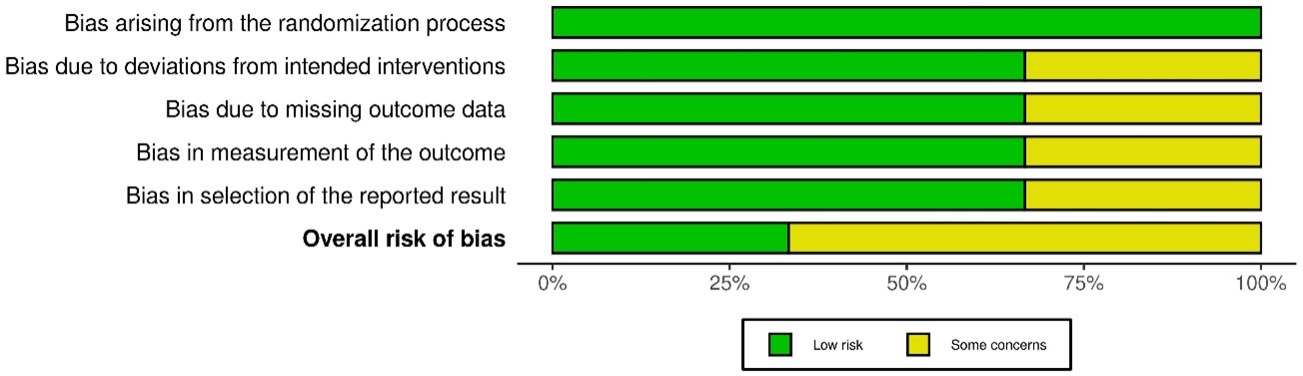
Summary plot: RoB assessment using Cochrane Rob2 tool for randomized controlled trial (*n* = 3).

For the three RCTs [24, 27, 28], the Cochrane RoB2 tool was used (Figures 4 and 5). All trials described their randomization processes; however, only Liu et al. [24] reported allocation concealment. The absence of this detail in the other trials increased the risk of selection bias. Blinding emerged as a major limitation; Maksoud Nassar et al. [27] and Koc [28] were open‐label studies, and Liu et al. [24] applied single blinding (participants only), increasing performance and detection bias. The attrition rate was low across all trials (> 90% retention). However, Maksoud Nassar et al. [27] reported a slightly higher dropout rate in one treatment arm. The selective reporting risk was generally low, but Liu et al. [24] lacked pre‐specified secondary outcomes. Based on the RoB2 assessment, Koc [28] demonstrated the highest‐quality RCT (low risk except for blinding), and Maksoud Nassar et al. [27] were rated at moderate risk, mainly because of its open‐label design. The detailed domain‐level assessments for randomized controlled trials (RoB 2) and non‐randomized studies (MINORS) are provided in Tables S2 and S3.

### 3.2 Therapeutic Interventions and Treatment Protocols

Therapeutic interventions (Table 2) were categorized into distinct pharmacological classes. Recent investigations prominently included monoclonal antibody therapies. Lovato et al. [26] used subcutaneous erenumab at a monthly dose of 140 mg, and Hoskin and Fife [20] evaluated fremanezumab and galcanezumab regimens. Beta‐blocker protocols focused on propranolol. Çelik et al. [22] implemented weight‐adjusted dosing ranging from 20 to 60 mg daily, and Salmito et al. [23] utilized fixed daily doses of 40–80 mg.

**TABLE 2:** Therapeutic interventions and treatment protocols across studies.

Study ID	Intervention(s)	Route	Dose (mean ± SD or range)	Control group	Treatment duration
Hoskin and Fife, 2022 [20]	Erenumab (11), Fremanezumab (9), Galcanezumab (6)	Subcutaneous	N/A	N/A	Not reported
Iwasaki et al., 2007 [21]	Lomerizine (22), Dihydroergotamine (2), Paroxetine (1), Dimetetiazine (2), etc.	Oral	Lomerizine: 10 mg/day	N/A	1–2 months
Çelik et al., 2020 [22]	Propranolol	Oral	20 mg, after 1 month was increased to 40 mg or 60 mg twice a day in patients ≤ 60 kg or > 60 kg in body weight, respectively	N/A	Not reported
Salmito et al., 2017 [23]	Amitriptyline Flunarizine Nortriptyline Propranolol Topiramate Valproate Venlafaxine	Oral	Amitriptyline: 25 mg or 50 mg/day Flunarizine: 10 mg/day Nortriptyline: 50 mg/day Propranolol: 40 mg or 80 mg/day Topiramate: 100 mg or 200 mg/day Valproate: 500 mg or 1000 mg/day Venlafaxine: 75 mg/day	N/A	3 months
Liu et al., 2017 [24]	Venlafaxine, Flunarizine, Valproic acid	Oral	Venlafaxine: 37.5 mg/day, Flunarizine: 10 mg/day Valproic acid: 500 mg BID	Active comparators	3 months
Baier et al., 2009 [25]	Beta‐blockers (67%), Valproic acid (8%), Topiramate (8%), Butterbur root (5%)	Oral	Metoprolol: 150 mg (50–250); Propranolol: 160 mg (40–240)	Conservative therapy (14/26)	23 ± 15 months
Lovato et al., 2023 [26]	Erenumab	Subcutaneous	Erenumab: 140 mg	Propranolol (40–80 mg), Flunarizine (5 mg)	mAb: 26.4 weeks; Conv: 28.3 weeks
Maksoud Nassar et al., 2023 [27]	Cinnarizine, Propranolol, Topiramate	Oral	Cinnarizine: 150 mg/day Propranolol: 40 mg/day Topiramate: 25 mg/day	N/A	3 months
Koc, 2024 [28]	Citalopram, Amitriptyline	Oral	Citalopram: 20 mg/day Amitriptyline: 10 mg/day	N/A	6 months

Abbreviations: BID, twice daily; Conv, conventional treatment; mAb, monoclonal antibody; mg, milligrams; mg/day, milligrams per day; N/A, not available or not applicable; SD, standard deviation.

Calcium channel blockers included flunarizine (5–10 mg daily) in a Chinese trial by Liu et al. [24] and cinnarizine (150 mg daily) in an Egyptian study by Maksoud Nassar et al. [27] Antidepressants included amitriptyline (10–50 mg daily) and venlafaxine (37.5–75 mg daily). Anticonvulsant options included topiramate (25–200 mg daily) and valproic acid (500–1000 mg daily) [23, 27]. Treatment durations ranged from 1 to 2 months in earlier investigations to up to 6 months in more recent protocols. Monoclonal antibodies were administered in 28‐day cycles.

### 3.3 Clinical Outcomes, Efficacy, and Safety

Clinical outcomes (Table 3) demonstrated consistent therapeutic benefits across multiple validated metrics. The anti‐CGRP monoclonal antibody class demonstrated particularly interesting results in a retrospective cohort study by Hoskin and Fife [20], in which erenumab 140 mg monthly resulted in significant symptom improvement in 27.3% (3/11) of patients, moderate improvement in 27.3% (3/11), and mild improvement in 18.2% (2/11). Fremanezumab showed a slightly less robust response profile, with 22.2% (2/9) of patients reporting significant improvement [20]. Galcanezumab emerged as the most effective treatment, with 50% (3/6) achieving significant improvement and only 16.7% (1/6) showing no response. However, the small sample size necessitated cautious interpretation.

**TABLE 3:** Clinical outcomes and reported adverse events across included studies.

Study (author, year)	Primary outcome(s)	Measurement tool(s)	Key findings	Adverse events
Hoskin and Fife 2022 [20]	Improvement in VM symptoms	Study‐specific improvement scale	Erenumab: Significant (3), moderate (3), mild (2), no improvement (1). Fremanezumab: Significant (2), moderate (2), mild (3), no improvement (2). Galcanezumab: Significant (3), moderate (1), mild (1), no improvement (0). Ubrogepant: Significant (1), no improvement (1)	Not reported
Iwasaki et al. 2007 [21]	Improvement in vertigo/dizziness symptoms	Study‐specific improvement scale	Lomerizine: 14 resolved, 5 well‐controlled, 3 poorly controlled Other drugs: 2 resolved, 3 well‐controlled, 3 poorly controlled	5/33 (15%) reported side effects (unspecified)
Çelik et al. 2020 [22]	Efficacy of propranolol; improvement in VM symptoms	VAS, DHI, VSS, VADL	VSS: Pre: 26.47 ± 7.97 → Post: 4.84 ± 8.24 DHI: Pre: 50.21 ± 22.39 → Post: 9.31 ± 9.86 VADL: Pre: 186.63 ± 79.65 → Post: 55.52 ± 51.89 Vertigo frequency: Reduced from twice daily to once every 2 months	Not reported
Salmito et al. 2017 [23]	Improvement in VM symptoms; global improvement in vestibular/migraine symptoms	VAS, VSS	VAS (vertigo): Overall pre: 7.04 → post: 3.05 (*p* < 0.001) VAS (headache): Overall pre: 7.87 → post: 3.32 (*p* < 0.001)	Not reported
Liu et al. 2017 [24]	Improvement in VM symptoms; DHI domains; VSS; vertigo attack frequency	DHI, VSS	VSS: Venlafaxine (5.96 → 3.78), Flunarizine (6.41 → 5.86), Valproic acid (5.8 → 5.3) DHI: Venlafaxine (41.74 → 31.3), Flunarizine (46.64 → 39.82), Valproic acid (46.8 → 38.7) Vertigo frequency: Venlafaxine (5.83 → 3.09), Flunarizine (4.95 → 4.15), Valproic acid (5.1 → 2.35)	Venlafaxine: Nausea (2), insomnia (1), palpitations (1), lethargy (1) Flunarizine: Somnolence (5), nausea (1) Valproic acid: Nausea (2), somnolence (1), indigestion (1)
Baier et al. 2009 [25]	Intensity, duration, frequency of vertigo attacks	Not specified	Vertigo frequency: Pre: 7.82 ± 1.08 → post: 3.82 ± 1.08 Headache frequency: Pre: 8.31 ± 3.55 → post: 2.88 ± 1.26 Duration of symptoms: Pre: 31.01 ± 8.24 h → post: 12.58 ± 2.55 h	Not reported
Lovato et al. 2023 [26]	Migraine days; vestibular function	DHI	DHI: Pre: 30.2 ± 7.2 → post: 8.1 ± 3.1 Migraine days (last 3 months): Pre: 12.4 → post: 5.1	0/23 reported side effects
Maksoud Nassar et al. 2023 [27]	Duration/frequency/severity of headache/vertigo attacks	DHI, CDP, SOT	DHI: Cinnarizine (−0.3 ± 0.2), Propranolol (−0.4 ± 0.2), Topiramate (−0.5 ± 0.2) Vertigo frequency: Cinnarizine (−0.43 ± 0.39), Propranolol (−0.53 ± 0.31), Topiramate (−0.55 ± 0.34) Headache frequency: Cinnarizine (−0.17 ± 0.30), Propranolol (−0.43 ± 0.36), Topiramate (−0.55 ± 0.16)	Cinnarizine: 2/15 (GI symptoms) Propranolol: 1/15 (hypotension). Topiramate: 3/15 (numbness, weight loss)
Koc 2024 [28]	Vertigo attack frequency; symptom improvement	DHI	DHI: Pre: 52.26 ± 20.19 → post: 12.29 ± 8.93 Vertigo frequency: Pre: 4.0 ± 0.4 → post: 0.4 ± 0.1	Citalopram: 2/152 (unspecified) Amitriptyline: 0/152

Abbreviations: CDP, computerized dynamic posturography; DHI, dizziness handicap inventory; SOT, sensory organization test; VADL, vestibular disorder activities of daily living scale; VAS, visual analog scale; VSS, vertigo symptom scale.

Calcium channel blockers exhibited dose‐dependent and compound‐specific variability. Lomerizine at 10 mg/day [21] achieved complete symptom resolution in 63.6% (14/22) of patients versus only 28.6% (2/7) resolution with other medications (dihydroergotamine and paroxetine). Cinnarizine 150 mg/day showed more modest DHI score reductions of −0.3 ± 0.2 points compared with propranolol [27]. Flunarizine performance varied significantly between studies. At a dose of 10 mg/day, it reduced VSS scores from 6.41 ± 1.99 to 5.86 ± 1.55 (Δ‐0.55) [24], whereas at 5 mg/day, it decreased vertigo visual analog scale (VAS) scores from 6.82 to 4.56 (Δ‐2.26) [23]. These findings suggest a potential nonlinear dose–response relationship in VM management.

Beta‐blockers have demonstrated particularly positive results in certain domains. Propranolol at 40–60 mg/day produced remarkable improvements in VSS (26.47 ± 7.97 to 4.84 ± 8.24, Δ‐21.63) and DHI (50.21 ± 22.39 to 9.31 ± 9.86, Δ‐40.90) [22], while reducing vertigo attack frequency from twice daily to once every two months. However, Salmito et al. [23] reported more modest effects with propranolol at 40–80 mg/day, noting a reduction in VAS scores from 6.33–7.75 to 3.25–4.00.

The antidepressant class has yielded interesting results. Venlafaxine at 37.5 mg/day reduced vertigo frequency from 5.83 ± 3.20 to 3.09 ± 1.68 attacks/month (Δ‐2.74) and improved DHI scores from 41.74 ± 16.90 to 31.30 ± 14.14 (Δ‐10.44) [24]. Citalopram at 20 mg/day in a large sample (*n* = 152) demonstrated significant DHI improvements from 52.26 ± 20.19 to 12.29 ± 8.93 (Δ‐39.97) and reduced vertigo attacks from 4.0 ± 0.4 to 0.4 ± 0.1 per month, suggesting potent serotonergic modulation of VM pathways [28].

The use of antiepileptic drugs yielded conflicting results. Valproic acid at 500 mg twice daily had minimal impact on VSS scores (5.80 ± 1.82 to 5.30 ± 1.08, Δ‐0.50) [24]. However, topiramate at 25–200 mg/day [23, 27] reduced headache frequency by −0.55 ± 0.16 attacks per month and decreased vertigo‐related VAS scores from 8.29 to 3.33 in the higher‐dose group studied by Salmito et al. [23].

The safety data revealed important considerations. Flunarizine caused somnolence in 22.7% (5/22) of patients [24], venlafaxine led to nausea in 8.7% (2/23), and topiramate was associated with numbness in 13.3% (2/15) and weight loss in 6.7% (1/15) [27]. Anti‐CGRP monoclonal antibodies produced no adverse events in 23 patients over a 26.4‐week period, suggesting an excellent safety profile [26]. Methodological heterogeneity across studies, including seven different primary outcome measures and varying treatment durations (1–6 months), complicates cross‐study comparisons. The outcomes highlight the consistently strong performance of propranolol across multiple metrics (VSS, DHI, and attack frequency) and the emerging potential of anti‐CGRP therapies.

The structured vote‐counting synthesis from the available pre/post data, β‐blockers as reported by Çelik 2020 [22] demonstrated improvement in both VSS and DHI, with substantial reductions from baseline. Antidepressants as reported by Koc 2024 [28] and CGRP monoclonal antibodies as reported by Lovato 2023 [26] each demonstrated improvement in DHI. No analyzable paired data were available for calcium channel blockers, antiepileptics, ergot derivatives, histamine analogues, or supplements. No study with analyzable data showed worsening or neutrality. The complete structured synthesis is shown in Table S4.

## 4 Discussion

This systematic review presents the existing evidence on five drug classes used for VM prophylaxis: anti‐CGRP monoclonal antibodies, antiepileptics, beta‐blockers, calcium channel blockers, and antidepressants. Although the heterogeneity of the included studies did not allow for quantitative synthesis, our qualitative analysis revealed important trends and clinical insights that warrant consideration of the drug classes with the highest potential.

### 4.1 Anti‐CGRP Monoclonal Antibodies

Anti‐CGRP monoclonal antibodies are a modern class of migraine prophylaxis. Agents such as erenumab, fremanezumab, and galcanezumab target CGRP, a key mediator in migraine pathophysiology. By blocking the CGRP ligand, these therapies reduce neurogenic inflammation, trigeminal activation, and vascular sensitization, all of which contribute to migraine and possibly vestibular symptoms [29].

In this review, Hoskin and Fife [20] observed a substantial improvement in symptoms following anti‐CGRP therapy. Galcanezumab provided significant relief in 50% of patients, whereas erenumab and fremanezumab resulted in positive but slightly lower response rates. Furthermore, Lovato et al. [26] demonstrated that erenumab significantly reduced migraine days (12.4 to 5.1) and DHI scores (30.2 to 8.1) over approximately 6 months, indicating improvements in both migraine and vestibular domains. No adverse events were reported, suggesting a favorable safety profile. In comparison, a recent prospective study reported even higher response rates: 90% of patients experienced a ≥ 50% reduction in vertigo, 86% in headache frequency, and 80% in Migraine Disability Assessment scores. Notably, 78% of participants showed simultaneous improvement across all three domains. The number of dizziness days per month significantly decreased, from 10.3 to 0.8 over 12 months, reinforcing the strong and lasting benefits of anti‐CGRP therapy [30].

Despite these significant findings, RCTs assessing the efficacy of anti‐CGRP monoclonal antibodies for VM remain lacking. However, their impact on migraine and vestibular symptoms supports their role as second‐line or adjunctive prophylactic options for patients unresponsive to traditional approaches.

### 4.2 Antiepileptic Drugs

Due to their neurostabilizing effects, antiepileptic drugs have been mainly used to prevent migraines. These agents may also benefit vestibular symptoms through the reduction of central hyperexcitability. Among pharmacological agents for headache, antiepileptics continue to demonstrate the most consistent efficacy. Topiramate, a carbonic anhydrase inhibitor, acts via several mechanisms, including voltage‐gated sodium channel blockade and facilitation of GABAergic inhibition [31]. In the comparative study by Maksoud Nassar et al. [27], topiramate was the most effective agent for reducing headache frequency (−0.55 ± 0.16/month) and vertigo (−0.55 ± 0.34/month). In the cohort treated by Salmito et al. [23], vertigo VAS scores significantly declined from 8.29 to 3.33. However, the use of topiramate may be limited by adverse events, including paresthesia (13.3%) and weight loss (6.7%), which may contraindicate its use in certain individuals.

Valproate demonstrated modest benefits on DHI and VSS scores in a study by Liu et al. [24] Given its efficacy–safety ratio and teratogenic potential, valproate is more appropriate for secondary or adjunctive use in VM. A pooled analysis of controlled trials showed that anticonvulsants as a class reduced migraine frequency by approximately 1.3 attacks per 28 days and more than doubled the likelihood of achieving a ≥ 50% reduction in attack frequency. Among these, topiramate and valproate showed efficacy, whereas agents such as lamotrigine and clonazepam did not demonstrate significant benefit [32].

Other reports in the literature have highlighted the effectiveness of anticonvulsants in obese patients and have identified lamotrigine as particularly beneficial for vertigo symptoms rather than headache. However, lamotrigine was not included in any of the studies analyzed in our review [33].

### 4.3 Beta‐Blockers

Propranolol and metoprolol are well‐established first‐line treatments for migraine prophylaxis. Their shared mechanisms, namely, regulation of sympathetic overactivity and stabilization of vascular tone in the vessels supplying the labyrinth and central vestibular pathways, seem to reduce the severity and frequency of vertigo attacks [34]. In a retrospective study by Salviz et al. [35], 65% of patients with VM experienced a notable improvement in vertigo episodes following propranolol use. Another study by Van Ombergen et al. [36] found a 50% reduction in vestibular symptoms among patients treated with metoprolol.

Despite their benefits, several concerns surround the use of beta‐blockers, including common adverse effects such as fatigue and exercise intolerance, a risk of bronchospasm in asthmatic patients, and the potential to exacerbate hypotension and bradycardia in susceptible individuals [37]. Furthermore, a large proportion of patients with VM are susceptible to psychiatric disorders, such as depression, which may be exacerbated by beta‐blocker therapy [38].

### 4.4 Calcium Channel Blockers

Flunarizine is the most promising calcium channel blocker for migraine prophylaxis, despite the limited supporting evidence. Its effectiveness may result from the suppression of cortical spreading depression (CSD), a wave of neuronal and glial depolarization that propagates across the cerebral cortex [39]. Activation of the trigeminal nerve by CSD may trigger inflammation in sensitive structures that induces migraine [40]. By blocking T‐type calcium channels, flunarizine may suppress CSD, and preliminary clinical trials have demonstrated encouraging outcomes. In an RCT, Liu et al. [24] reported a 70% reduction in vertigo attacks with flunarizine compared with placebo. Notably, an observational study by Rashid et al. [41] found that 90% of patients with VM taking flunarizine experienced symptomatic improvement, in contrast to only 32% of patients on other medications. Despite these findings, more evidence is needed to confirm the efficacy of calcium channel blockers for prophylaxis and to assess whether the benefits outweigh potential adverse effects, such as sedation due to antihistaminergic properties [42]. Future research is required to ascertain whether these are superior to other prophylactic treatment options.

### 4.5 Antidepressants

Psychiatric comorbidities such as depression and anxiety frequently coexist with VM, leading to frequent prescription of antidepressants, which may offer dual benefits in alleviating migraines. The role of serotonin in migraine pathophysiology involves several mechanisms, including its low levels potentially causing blood vessel dilation and triggering migraine onset [43]. Another possible mechanism, though not fully understood, is that reduced serotonin levels may activate the trigeminovascular nociceptive pathway, which transmits pain signals from the face and head [44]. Antidepressants that increase serotonin reuptake, such as selective serotonin reuptake inhibitors, may be beneficial for VM prophylaxis. Increasing norepinephrine levels, particularly through serotonin‐norepinephrine reuptake inhibitors (SNRIs), can modulate the activity of the locus coeruleus, which is involved in balance and spatial orientation through vestibular input [35]. Venlafaxine has shown promising results; an RCT by Salviz et al. [35] revealed a 70% response rate in patients with definite VM, a result comparable with the beta‐blocker treatment group.

A systematic review and meta‐analysis by Xu et al. [45] found that patients treated with the tricyclic antidepressant amitriptyline were more likely to achieve a ≥ 50% reduction in headache burden compared with those receiving placebo. This efficacy was similar to that observed with selective serotonin reuptake inhibitors and serotonin‐norepinephrine reuptake inhibitors. However, clinical data evaluating antidepressants for VM prophylaxis remain limited. Potential adverse effects, including the anticholinergic properties of tricyclic antidepressants and the risk of hypertension or serotonin syndrome with serotonin‐norepinephrine reuptake inhibitors, must be weighed against their potential benefits for patients with VM [46].

### 4.6 Vestibular Rehabilitation Therapy

Although none of the included studies focused solely on VRT, the use of vestibular‐specific outcome metrics such as the DHI, VSS, and VADL scale implies an indirect evaluation of functional outcomes. Çelik et al. [22] reported a decrease in DHI scores from 50.21 to 9.31 and in VADL scores from 186.6 to 55.5 among patients receiving VRT with pharmacologic treatment, suggesting improved functional recovery with the addition of VRT. By facilitating central vestibular compensation, improving balance confidence, and reducing motion sensitivity, VRT serves as a valuable intervention during interictal phases or in addressing residual symptoms. VRT combined with pharmacotherapy may be advantageous, particularly for patients with prolonged or chronic VM symptoms. Koc and Akkılıc [47] also found that vestibular rehabilitation significantly improved DHI, VADL, dizziness frequency, and headache scores in both the VM and vestibular dysfunction groups, with posttreatment computerized dynamic posturography scores showing notable improvement from baseline.

### 4.7 Clinical Implications

The findings of this review show an increasing number of treatment options for VM, each with varying degrees of effectiveness and safety. Anti‐CGRP monoclonal antibodies offer promising benefits for both headache and vestibular symptoms, making them a potential second‐line or additional option for patients unresponsive to traditional treatments. Antiepileptic drugs, particularly topiramate, continue to be a key choice for VM prevention because of their consistent efficacy, although tolerability may be an issue for some individuals. Beta‐blockers and calcium channel blockers, such as flunarizine, also contribute to symptom control, especially in patients with underlying cardiac diseases. The increasing use of antidepressants highlights the need to address mental health concerns associated with VM. Furthermore, the addition of VRT to drug treatment may improve recovery, particularly for patients with ongoing symptoms. A personalized, team‐based approach is crucial to achieve the best results in VM management.

### 4.8 Limitations

This systematic review had several limitations that warrant consideration. First, substantial heterogeneity among the included studies limited the feasibility of direct comparisons and robust statistical analyses. Second, most data originated from retrospective observational studies, whereas the few RCTs used open‐label designs. Third, outcome reporting was inconsistent, with five studies omitting adverse events, which may have led to an underestimation of safety concerns. Fourth, the included studies did not distinguish between confirmed VM and probable VM (pVM) in their reporting. Since the outcomes were presented in a pooled manner, we were unable to analyze these groups separately. This lack of stratification may have influenced the observed effects and should be taken into account when interpreting the generalizability of our findings. Another limitation of this review is the restricted database coverage. Since we did not have institutional access to Embase or Scopus, our search was limited to PubMed, Web of Science, and Google Scholar. Although we supplemented the search with detailed Boolean strategies and multiple synonyms, the lack of access to additional databases may have led to missed studies and potential publication bias, which should be considered when interpreting our findings. Finally, the absence of biomarker investigations hindered deeper insights into the efficacy of various therapies. These limitations highlight the need for large‐scale, standardized RCTs with validated endpoints to clarify the role, efficacy, and safety profiles of prophylactic treatments for VM.

## 5 Conclusion

This systematic review evaluated the prophylactic management of VMs, focusing on both pharmacological and non‐pharmacological interventions. Among pharmacological treatments, propranolol and topiramate emerged as effective first‐line prophylactic treatments, supported by moderate‐quality evidence demonstrating significant reductions in VM frequency and severity. Additionally, anti‐CGRP monoclonal antibodies, such as erenumab and galcanezumab, showed promising efficacy and tolerability, particularly in patients with treatment‐refractory VMs. Non‐pharmacological interventions, including vestibular rehabilitation, also provided functional benefits and should be integrated as adjunctive therapies. Other agents, including calcium channel blockers and antidepressants, presented variable outcomes, highlighting the importance of individualized treatment approaches. Interpretation of these findings is limited by methodological heterogeneity, a predominance of retrospective studies, and inconsistencies in outcome reporting. Addressing these limitations requires well‐designed, large‐scale RCTs using standardized diagnostic criteria and outcome measures. Future research should explore the long‐term safety and efficacy of emerging therapies, the potential benefits of combination treatment approaches, and the role of predictive biomarkers to facilitate individualized VM management. Although significant progress has been made in understanding and managing VMs, further research is necessary to refine and optimize treatment strategies.

## Author Contributions

The authors have reviewed and approved the final manuscript for submission, acknowledging their responsibility for the accuracy and integrity of the content presented. Each author has contributed equally to the development and execution of this research, applying their expertise and effort to bring it to completion.

## Ethics Statement

The authors have nothing to report.

## Consent

The authors have nothing to report.

## Conflicts of Interest

The authors declare no conflicts of interest.

## Supporting information


**Table S1:** Search strategy and restrictions (PubMed, Web of Science, Google Scholar).
**Table S2:** Cochrane RoB 2 domain‐level bias assessment for randomized controlled trials.
**Table S3:** MINORS assessment for non‐randomized studies.
**Table S4:** Structured vote‐counting synthesis of pharmacological interventions for vestibular migraine.

## Data Availability

Data sharing not applicable to this article as no datasets were generated or analyzed during the current study.
