# Supplementary material for: Prophylactic Management of Vestibular Migraine: A Systematic Review
Source: Ann Clin Transl Neurol. 2025 Oct 30;12(12):2384–97. doi: 10.1002/acn3.70234 (PMC12698959; doi:10.1002/acn3.70234)
Supplement: Supplementary file 1 — Table S1: Search strategy and restrictions (PubMed, Web of Science, Google Scholar). Table S2: Cochrane RoB 2 domain‐level bias assessment for randomized controlled trials. Table S3: MINORS assessment for non‐randomized studies. Table S4: Structured vote‐counting synthesis of pharmacological interventions for vestibular migraine. [file ACN3-12-2384-s001.docx]

**Supplementary Material**

**Table S1.** Search strategy and restrictions

| **Database** | **Search date** | **Detailed search string** | **Restrictions applied** |
| --- | --- | --- | --- |
| PubMed | April 2025 | (“vestibular migraine” OR “vestibular migrain” OR “migrainous vertigo” OR “migraine-associated vertigo” OR “migraine-associated dizziness” OR “migraine-anxiety-associated dizziness” OR “migraine-related vestibulopathy” OR “vestibular dysfunction” OR “vestibular symptoms”) AND (“beta-blocker” OR “beta blocker” OR “antiseizure medication” OR “anticonvulsants” OR “anti-epileptic” OR “antiepileptic drugs” OR “antiepileptic agents” OR “calcium channel blockers” OR “tricyclic antidepressants” OR “tricyclics” OR “serotonin-norepinephrine reuptake inhibitors” OR “SNRI” OR “vestibular rehabilitation” OR “physical therapy” OR “rehabilitative therapy” OR “erenumab” OR “fremanezumab” OR “galcanezumab”) AND (“acetazolamide” OR “lamotrigine” OR “topiramate” OR “valproic acid” OR “verapamil” OR “diltiazem” OR “lomerizine” OR “flunarizine” OR “adrenergic beta-antagonists” OR “propranolol” OR “atenolol” OR “metoprolol” OR “gabapentin” OR “venlafaxine” OR “benzodiazepines” OR “lorazepam” OR “diazepam” OR “dimenhydrinate” OR “antiemetics” OR “histamine antagonists” OR “antihistamines” OR “meclizine” OR “betahistine” OR “cyproheptadine” OR “promethazine” OR “sumatriptan” OR “rizatriptan” OR “pizotifen” OR “migraine medications”) AND (“randomized controlled trial” OR “controlled clinical trial” OR “randomized” OR “placebo-controlled” OR “drug therapy” OR “clinical trial” OR “random assignment” OR “trial” OR “study groups” OR “cohort study” OR “retrospective study” OR “prospective study” NOT (“animals” NOT “humans”)) | Limited to articles in English |
| Web of Science | April 2025 | (“vestibular migraine” OR “migrainous vertigo” OR “migraine-associated dizziness” OR “vestibular dysfunction” OR “vestibular symptoms”) AND (“beta-blocker” OR “beta blocker” OR “antiseizure medication” OR “anticonvulsants” OR “calcium channel blockers” OR “tricyclic antidepressants” OR “serotonin-norepinephrine reuptake inhibitors” OR “vestibular rehabilitation” OR “physical therapy” OR “erenumab” OR “fremanezumab”) AND (“acetazolamide” OR “lamotrigine” OR “topiramate” OR “valproic acid” OR “verapamil” OR “propranolol” OR “gabapentin” OR “venlafaxine” OR “antiemetics” OR “meclizine” OR “betahistine” OR “sumatriptan” OR “rizatriptan”) AND (“randomized controlled trial” OR “controlled clinical trial” OR “drug therapy” OR “clinical trial” OR “prospective study” OR “cohort study” NOT (“animals”)) | Limited to articles in English |
| Google Scholar | April 2025 | Same search concepts as above were applied in simplified form to capture additional grey literature (e.g., “vestibular migraine” AND “treatment” AND “clinical trial”). Due to platform limitations, full Boolean expansion was not feasible. | Limited to articles in English |

**Table S2.** Cochrane RoB 2 domain-level bias assessment for randomized controlled trials.

| **Authors/Year** | **Study Type** | **D1: Randomization Process** | **D2: Deviations from Intended Interventions** | **D3: Missing Outcome Data** | **D4: Outcome Measurement** | **D5: Selection of Reported Result** | **Overall Risk** |
| --- | --- | --- | --- | --- | --- | --- | --- |
| Liu, 2017 | RCT | Low | Unclear | Unclear | Low | Unclear | Unclear |
| Nassar, 2023 | RCT | Low | Low | Low | Unclear | Low | Unclear |
| Koc, 2024 | RCT | Low | Low | Low | Low | Low | Low |

**Table S3.** MINORS Assessment for Non-Randomized Studies

| **Study ID** | **1. Aim** | **2. Consecutive Patients** | **3. Prospective Data** | **4. Endpoints Appropriate** | **5. Unbiased Assessment** | **6. Adequate Follow-up** | **7. <5% Loss to Follow-up** | **8. Sample Size Calculation** | **9. Control Group** | **10. Contemporary Groups** | **11. Baseline Equivalence** | **12. Statistical Analyses** | **Total Score** |
| --- | --- | --- | --- | --- | --- | --- | --- | --- | --- | --- | --- | --- | --- |
| Hoskin, 2022 | 2 | 1 | 0 | 2 | 1 | 1 | 1 | 0 | N/A | N/A | N/A | N/A | 8/16 |
| Iwasaki, 2007 | 2 | 1 | 0 | 2 | 1 | 1 | 2 | 0 | N/A | N/A | N/A | N/A | 9/16 |
| Çelik, 2020 | 2 | 1 | 0 | 2 | 1 | 1 | 2 | 2 | N/A | N/A | N/A | N/A | 11/16 |
| Salmito, 2016 | 2 | 1 | 0 | 2 | 1 | 1 | 2 | 0 | N/A | N/A | N/A | N/A | 9/16 |
| Baier, 2009 | 2 | 1 | 0 | 2 | 1 | 1 | 2 | 0 | 1 | 1 | 2 | 2 | 14/24 |
| Lovato, 2023 | 2 | 1 | 0 | 2 | 1 | 1 | 2 | 0 | 1 | 1 | 2 | 2 | 12/24 |

**Table S4.** Structured vote-counting synthesis of pharmacological interventions for vestibular migraine.

| **Intervention class** | **Outcome** | **Studies with Improvement** | **Studies with No Change** | **Studies with Worsening** |
| --- | --- | --- | --- | --- |
| **β-blocker** | VSS | 1 (Çelik 2020) | 0 | 0 |
|  | DHI | 1 (Çelik 2020) | 0 | 0 |
| **Antidepressant** | DHI | 1 (Koc 2024) | 0 | 0 |
| **CGRP monoclonal antibody** | DHI | 1 (Lovato 2023) | 0 | 0 |
| **Other classes** (e.g., calcium channel blockers, antiepileptics, ergot derivatives, histamine analogues, supplements) | All reported outcomes | No eligible paired pre–post data available | – | – |

**Abbreviations:** DHI: Dizziness Handicap Inventory, VSS: Vertigo Symptom Scale
